# Clinical results of the re-fixation of a Chevron olecranon osteotomy using an intramedullary cancellous screw and suture tension band

**DOI:** 10.1007/s11751-015-0211-9

**Published:** 2015-02-20

**Authors:** Marc L. Wagener, Marleen Dezillie, Yvette Hoendervangers, Denise Eygendaal

**Affiliations:** 1 Department of Orthopaedics, Rijnstate Ziekenhuis, Pb 9555, 6800 TA Arnhem, The Netherlands; 2 Department of Orthopaedics, AZ Groeninge, Kortrijk, Belgium; 3 Department of Surgery, St Antonius Hospital, Nieuwegein, The Netherlands; 4 Upper Limb Unit, Department of Orthopaedics, Amphia Hospital, Pb 90157, 4800 RL Breda, The Netherlands

**Keywords:** Chevron osteotomy, Re-fixation, Tension band

## Abstract

Exposure of the distal humerus in case of an articular fracture is often performed through a Chevron osteotomy of the olecranon. Several options have been described for re-fixation of the Chevron osteotomy. Pull-out of the hard-wear is often seen as complication. In this study, an evaluation of the re-fixation of the Chevron osteotomy through a cancellous screw and suture tension band was performed. The data of 19 patients in whom a Chevron osteotomy was re-fixated with a cancellous screw in combination with a suture tension band were used. Evaluation was performed by assessment of the post-operative X-rays and documentation of complications. In all 19 cases, evaluation of the post-operative X-rays showed complete consolidation without dislocation or other complications. Re-fixation of a Chevron osteotomy of the olecranon with a large cancellous screw with a suture tension band provides adequate stability to result in proper healing of the osteotomy in primary cases when early post-operative mobilisation is allowed. Complications as pull-out of the hard-wear were not reported.

## Introduction

Adequate exposure is essential in order to achieve anatomical reconstruction of the articular surface in case of an intra-articular distal humeral fracture. Several surgical approaches of the distal humerus, such as a transverse osteotomy of the olecranon, a triceps-reflecting approach, or a triceps-preserving approach [1–5], have been described. The posterior approach through a Chevron osteotomy of the olecranon is most often used and allows excellent visualisation of the distal humerus [3, 6].

For the re-fixation of the Chevron osteotomy, different fixation techniques have been described. In most studies, Kirschner wires (K-wires) with a metal tension band construction are used, less often fixation with a plate and screws, or an intramedullary cancellous screw with or without a tension band has been described. The philosophy of the tension band principle is that loading of the triceps results in compression forces across the osteotomy [7–9], although recent biomechanical studies could not support this theory [10, 11]. K-wires with a tension band construction are related to a complication rate of up to 80 % [3, 12–14]. Pull-out of the wires with chance of infection or secondary dislocation is most often seen. Fixation with a plate is also related to complications such as skin necrosis and secondary surgery for plate removal due to the bulkiness of the plate. This, together with economical reasons, makes a plate less popular for fixation of an osteotomy of the olecranon [15].

In a recent biomechanical study [11], three different methods for re-fixating the Chevron osteotomy of the olecranon were evaluated using Roentgen stereophotogrammetric analysis; bi-cortically placed K-wires with a tension band, a large cancellous screw with a tension band, and a large cancellous screw alone. The results showed that when a tension band is added to the fixation with a cancellous screw, the re-fixated Chevron osteotomy is able to withstand significant higher forces applied to the triceps compared to the cancellous screw alone. This allows early post-operative movement of the elbow more safely. Clinical evaluation of the re-fixation techniques from this study is essential.

In this multi-centre study, we evaluated the re-fixation of the Chevron osteotomy with a cancellous screw combined with a suture tension band and the associated complication rate. Evaluation of the re-fixation was performed by assessment of the post-operative X-rays and documentation of complications.

## Materials and methods

Patient data of two elbow reference centres were used. For this retrospective study, all patients in whom a Chevron osteotomy of the olecranon was re-fixated with a cancellous screw with tension band were included. Revision cases were excluded. In six consecutive years (2008–2013), 19 elbows in 19 patients (12 female, seven male) were found that met the criteria. The mean age of the patients at time of surgery was 62(20–91). The right elbow was involved in nine cases, the left elbow in 10. Two consultant upper limb surgeons performed all surgeries. In all patients, the indication for performing a Chevron osteotomy was the exposure of an intra-articular fracture of the distal humerus. In all cases, reposition and internal fixation of the fracture of the distal humerus were performed.

### Surgical technique

The following technique was used in all 19 cases: the Chevron osteotomy was performed as described by the Arbeitsgemeinschaft für Osteosynthesefragen(AO) foundation [3]. The apex distal Chevron osteotomy was performed using an oscillating saw. The osteotomy entered the joint at the depth of the trochlear notch. The osteotomy was reconstructed using a large cancellous screw with a high-strength suture tension band. Temporary reduction in the osteotomy was performed using two reduction clamps. A 4.5-mm drill was used to pre-drill the proximal part of the olecranon, and the intramedullary canal of the ulna was drilled with a 3.2-mm drill. Tapping was performed, and the length of the screw was determined by measuring the length of the tap when adequate fixation was achieved in the ulna. A 4.5-mm cancellous screw was placed. A washer was only added in elbows with poor bone quality. Tension band wiring was performed using Fibrewire (Arthrex, Naples, FL). The high-strength suture tension band was passed through a hole in the ulna approximately 4 cm distal to the osteotomy, passed over the cancellous screw and under the triceps tendon in a figure of eight, and then tied firmly. All elbows were allowed to start active and passive movement immediately after surgery.

### Evaluation of re-fixation

The re-fixation of the Chevron osteotomy was assessed using standard X-rays in AP and lateral view. The X-rays were assessed for consolidation of the osteotomy and for dislocation of the proximal fragment. All X-rays were evaluated independently by two of the authors (DE and YH). Patient data were scored for post-operative complications.

## Results

The mean time of follow-up was 14 months (range 2–42 months). There was only one patient with minimal follow-up of 2 months. Due to her age-related physical and mental status, additional hospital visits were not performed. The radiograph after 2 months showed proper consolidation of the osteotomy. Minimal follow-up of the rest of the patients included was 6 months.

The average screw length that was inserted was 89 mm (range 82–110 mm). In all 19 cases, evaluation of the post-operative X-rays showed complete healing of the osteotomy without dislocation. In all cases, no complication, such as infection, pull-out of the screw, or skin necrosis was noticed during follow-up. In none of these cases, hardware removal was necessary.

## Discussion

Several studies have been performed to analyse the stability of different fixation techniques of olecranon fractures and olecranon osteotomies [9, 11, 14, 16–19]. The Chevron osteotomy is most often used for the exposure of the distal humerus. Theoretically, it provides more intrinsic rotational stability than a transverse or an oblique osteotomy. It provides a larger area of cancellous bone contact, which probably results in improved bone healing in comparison with a transverse or an oblique osteotomy. In our opinion, absolute anatomical reduction in the osteotomy of the olecranon is essential to achieve the best functional outcome.

Since early mobilisation of the elbow after surgery of a distal humeral fracture is essential to regain as much function as possible, the re-fixated Chevron osteotomy should be able to withstand the high forces generated across the elbow in daily living in order to allow direct active movement of the elbow.

Classically, K-wires and metal tension band wiring were used for the fixation of simple olecranon fractures, 21.B1 according to the AO classification [20] (20) [20], and osteotomies of the olecranon. Due to the high complication incidence [2, 3, 12–14], such as pull-out of the hardware (reported up to 80 %), and subsequent defects of the skin and infection, nowadays alternative methods are available. As mentioned in the introduction, a recent biomechanical study [11] by the authors showed that when a tension band is added to the re-fixation of a Chevron osteotomy with a cancellous screw, the re-fixated Chevron osteotomy is able to withstand significant higher forces applied to the triceps compared to the cancellous screw alone. If the osteotomy is fixated with a cancellous screw alone, significant rotation and translation of the proximal part of the osteotomy starts to occur when it is loaded with a force of 350 N or more [11, 16]. In this study, a high-strength suture was used as a tension band instead of the metal wire tension band as used in the biomechanical study. Biomechanical research of Carofino et al. [21] showed that the biomechanical characteristics of high-strength suture tension bands are equivalent to metal wire tension bands.

Sané et al. [22] reported on the clinical results of fourteen elbows in which a transverse osteotomy of the olecranon was re-fixated with Kirschner wires and tension band.

In these 14 elbows, they found nine mal-unions (64 %). This high percentage of mal-unions might be due to the fact that anatomical reduction in a V-shaped Chevron osteotomy is easier than anatomical reduction in a transverse osteotomy, and the Chevron-shaped osteotomy provides more intrinsic stability than a transverse osteotomy.

This study shows proper consolidation of all re-fixated Chevron osteotomies. It should be noticed that all included cases were primary surgeries. Whether re-fixation of the Chevron osteotomy with a screw and tension band shows proper bone healing in revision cases cannot be concluded. In the study of Ring et al. [3], 45 Chevron osteotomies were re-fixated with Kirschner wires and metal tension band wiring. Twenty patients had had a prior osteotomy of the olecranon, and 44 out of 45 osteotomies healed without loss of alignment. Worth mentioning is that despite the high percentage of proper consolidation of the Chevron osteotomy, they also reported a 27 % patients that required removal of the wires used to repair the olecranon. Coles et al. [23] used an intramedullary cancellous screw with washer and metal tension band wiring in patients in which a Chevron osteotomy was used for the approach of a distal humeral fracture. In 61 patients, no non-unions were seen. This supports our data that screw and tension band fixation of primary re-fixation of a Chevron osteotomy results in consolidation of the osteotomy. In contrast to our study, they reported that in 29 % of the cases hardware removal was performed. We think that this probably is the result of the local irritation of the bursa and the skin that is often seen as a result of the metal tension band wiring.

Limitations of the current study are that it is a retrospective study design without a control group. Ideally, additional patient-related factors such as smoking, body mass index, and the presence of osteoporosis should have been available, but these have not been scored in all cases. On the other hand, since all re-fixated osteotomies showed proper bone healing, no further conclusion could have been drawn regarding which patient-related factors increase the risk of a non-union or a mal-union of a re-fixated osteotomy. Furthermore, experienced elbow surgeons performed all surgeries, and this possibly positively influences the result of the surgery.

In conclusion, re-fixation of a Chevron osteotomy of the olecranon with a large cancellous screw with a high-strength suture tension band provides adequate stability to result in anatomical consolidation of the osteotomy when early post-operative mobilisation is allowed. Pull-out of or removal of the hardware is prevented using this technique reducing the number of complications of a re-fixation.
